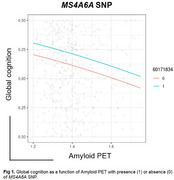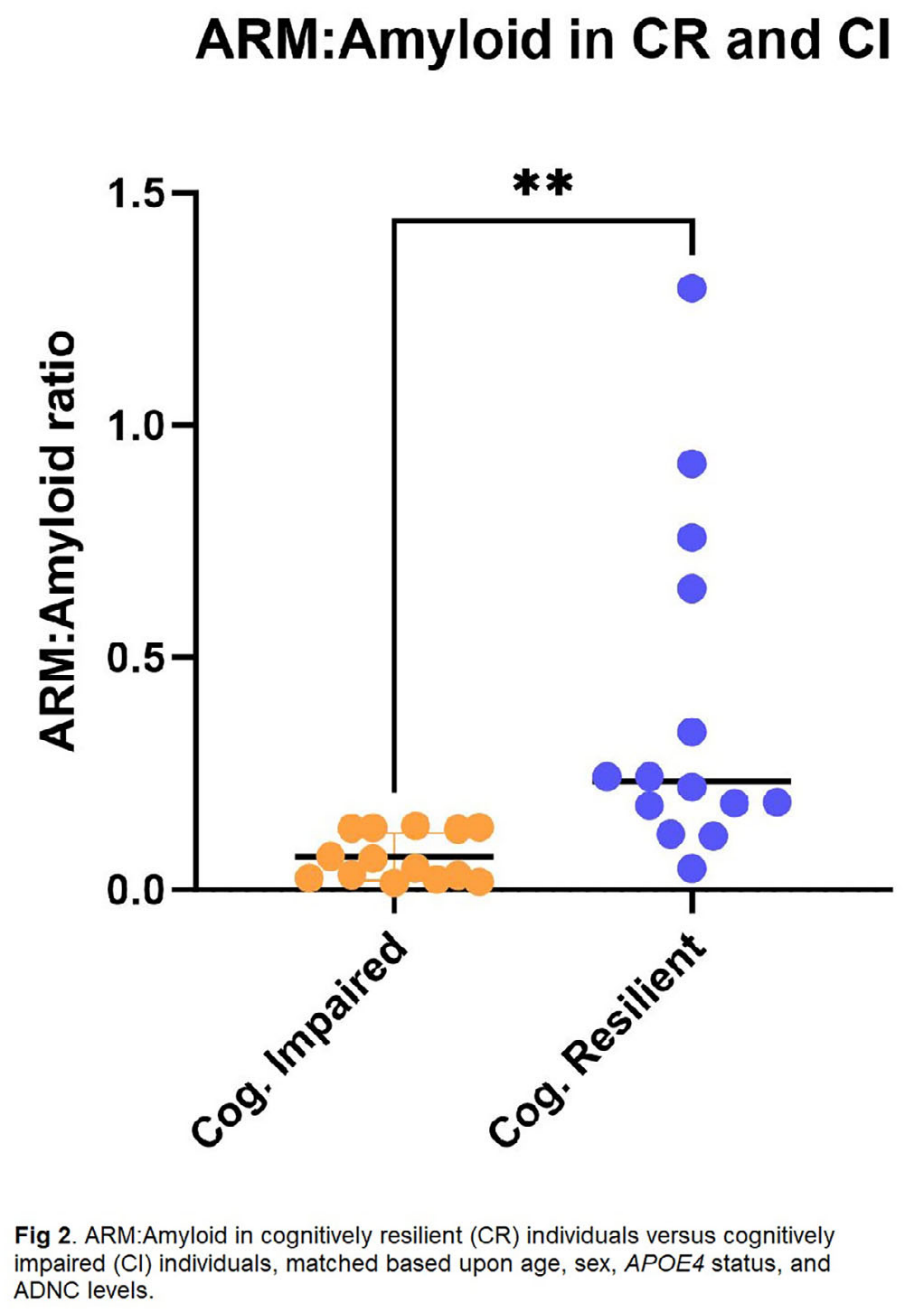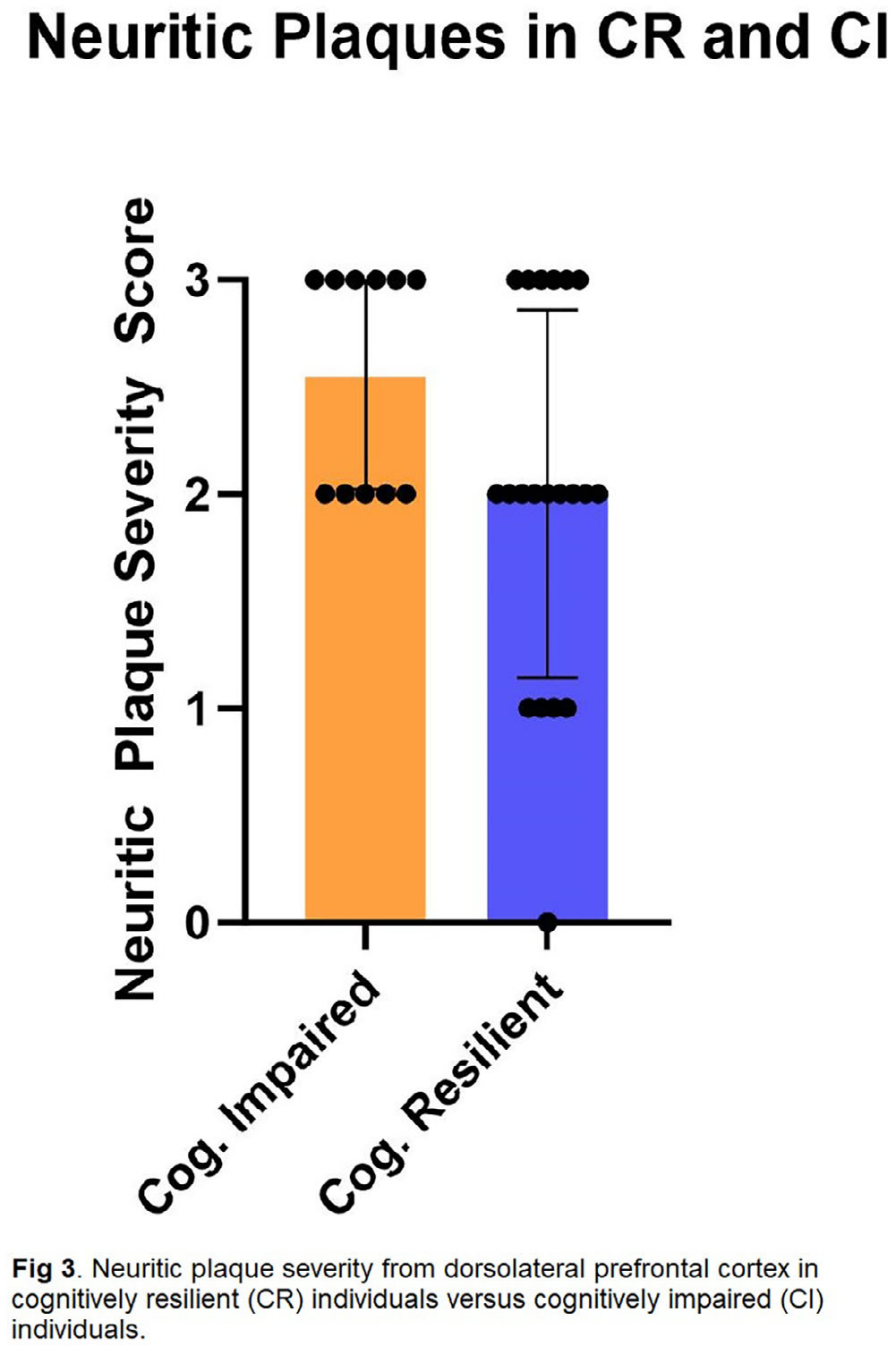# Cognitively resilient individuals show enrichment for *MS4A* gene SNPs and postmortem amyloid‐responsive microglia

**DOI:** 10.1002/alz70855_106867

**Published:** 2025-12-24

**Authors:** Aivi T. Nguyen, Vijay K. Ramanan, Scott A. Przybelski, Ekaterina I. Hofrenning, Ronald Petersen, Jonathan Graff‐Radford, David S. Knopman, Clifford R. Jack, R. Ross Reichard, Prashanthi Vemuri

**Affiliations:** ^1^ Department of Laboratory Medicine and Pathology, Mayo Clinic, Rochester, MN, USA; ^2^ Mayo Clinic, Rochester, MN, USA; ^3^ Department of Quantitative Health Sciences, Mayo Clinic, Rochester, MN, USA; ^4^ Department of Neurology, Mayo Clinic, Rochester, MN, USA; ^5^ Department of Radiology, Mayo Clinic, Rochester, MN, USA

## Abstract

**Background:**

Cognitive resilience is the discrepancy in individuals with intermediate to high Alzheimer's disease neuropathologic change (ADNC) and normal cognition. Currently, the role of microglial activation in cognitive resilience is understudied.

**Method:**

In the MCSA brain bank, high‐cognitively unimpaired (high‐CU) participants were defined as those performing in the upper quartile of their respective age groups based upon antemortem global cognition z‐scores, computed from memory, language, executive function, and visuospatial domain z‐scores. From the high‐CU group (*N* = 118, global z‐scores from 1.55 to 1.78), cognitively resilient (CR) individuals were identified as those with intermediate to high ADNC upon neuropathologic evaluation. First, regression models were fit to evaluate global cognition as a function of age, sex, amyloid PET, and microglial‐specific genes of interest, including *MS4A* gene cluster SNPs. Second, a subset of CR participants was analyzed for postmortem ARM expression in the dorsolateral prefrontal cortex. Single immunohistochemistry was performed on formalin‐fixed, paraffin‐embedded sections using antibodies against beta‐amyloid (6F3D) and CD163 (10D6). Digital image analysis was performed on scanned sections (40x, Leica Aperio GT 450), and ARM (CD163%area) and beta‐amyloid (%area) measurements were obtained using QuPath. Cognitively impaired (CI) control group was matched on age, sex, ADNC level, and *APOE4* status. Spearman correlations were performed between postmortem ARM and antemortem MRI, PET, plasma, and global cognition metrics.

**Result:**

A third (39/118) of high‐CU cases showed intermediate/high ADNC (CR group). Furthermore, 28% of CR individuals harbored an *MS4A6A* SNP, which showed a protective effect on cognition via amyloid‐PET (*p* = 0.043, Figure 1). Moreover, CR individuals harbored significantly higher ARM:amyloid than the CI group (*p* <0.001, Figure 2) and fewer neuritic plaques (*p* = 0.03, Figure 3). Antemortem white matter hyperintensities and global cognition were significantly correlated to ARM expression (r=0.556 [p=0.042] and r=0.648 [p=0.02], respectively).

**Conclusion:**

Microglial specific *MS4A6A* SNP, which modulates microglial activation, was seen in a third of CR individuals. Postmortem analyses in CR individuals revealed increased ARM:amyloid, which was also associated with higher antemortem cognitive performance. These findings underscore the role of microglial activation in cognitive resilience. Future work is needed to investigate microglial protective mechanisms and links between vascular disease and microglial activation.